# Monitoring and modifying brain oxygenation in patients at risk of hypoxic ischaemic brain injury after cardiac arrest

**DOI:** 10.1186/s13054-021-03678-3

**Published:** 2021-08-31

**Authors:** Markus Benedikt Skrifvars, Mypinder Sekhon, Erik Anders Åneman

**Affiliations:** 1 Department of Emergency Care and Services, Helsinki University Hospital and University of Helsinki, Helsinki, Finland; 2 Division of Critical Care Medicine, Department of Medicine, Faculty of Medicine, University of British Columbia, Vancouver, BC Canada; 3 Southwestern Clinical School, University of New South Wales, Sydney, NSW Australia; 4 Faculty of Medicine and Health Sciences, Macquarie University, Sydney, NSW Australia; 5 College of Health and Medicine, Australian National University, Canberra, NSW Australia; 6 Department of Anaesthesiology and Intensive Care Medicine, Institute of Clinical Sciences at Sahlgrenska Academy, University of Gothenburg, Gothenburg, Sweden; 7 Intensive Care Unit, Liverpool Hospital, South Western Sydney Local Health District, Liverpool, NSW Australia

## Abstract

This article is one of ten reviews selected from the Annual Update in Intensive Care and Emergency Medicine 2021. Other selected articles can be found online at https://www.biomedcentral.com/collections/annualupdate2021. Further information about the Annual Update in Intensive Care and Emergency Medicine is available from https://link.springer.com/bookseries/8901.

## Introduction

The majority of adverse clinical outcomes following successful resuscitation from cardiac arrest, are attributable to hypoxic ischemic brain injury [1]. The cornerstone of hypoxic ischemic brain injury management has traditionally focused on preventing secondary ischemic injury, following the return of spontaneous circulation (ROSC) [2]. Among the various mechanisms implicated in the pathophysiology of secondary injury, post-resuscitation cerebral ischemia is linked to central physiologic variables that may be modifiable [3]. Observational data demonstrate associations between perturbations in physiologic variables known to reduce cerebral blood flow (CBF)—such as arterial hypotension [4] and hypocapnia [5]—and adverse clinical outcome. This adds credence to the importance of optimizing cerebral oxygen delivery, to mitigate secondary ischemic injury. Recently, sentinel randomized controlled trials (RCTs) aimed at augmenting mean arterial pressure (MAP)—a key physiologic determinant of cerebral oxygen delivery—have yielded important insights into the importance of mitigating secondary cerebral ischemia [6, 7]. Although it did not establish a definitive link to improved neurological outcome, the COMACARE study demonstrated reduced levels of neurofilament light, a biomarker of brain injury, in patients undergoing an augmented MAP strategy following ROSC [8]. Patients may continue to experience episodes of brain hypoxia following cardiac arrest, despite goal-directed therapy and augmented MAP, with considerable heterogeneity in the underlying cerebrovascular hemodynamics in individual patients [9]. Thus, a targeted approach to the individualized management of hypoxic ischemic brain injury in the post-resuscitation phase requires the longitudinal monitoring of brain oxygenation—providing clinicians with real time physiologic data points to optimize cerebral oxygen delivery, similar to that applied in patients with traumatic brain injury (TBI) [10]. Near infrared spectroscopy (NIRS) provides an easily implemented and virtually complication-free way to monitor regional cerebral oxygen saturation (rSO2) in critically ill patients. The insertion of oxygen sensing catheters provides a real time assessment of the partial pressure of oxygen in brain tissue (PbtO2). This approach has gained widespread use following neurotrauma.

**Fig. 1:**
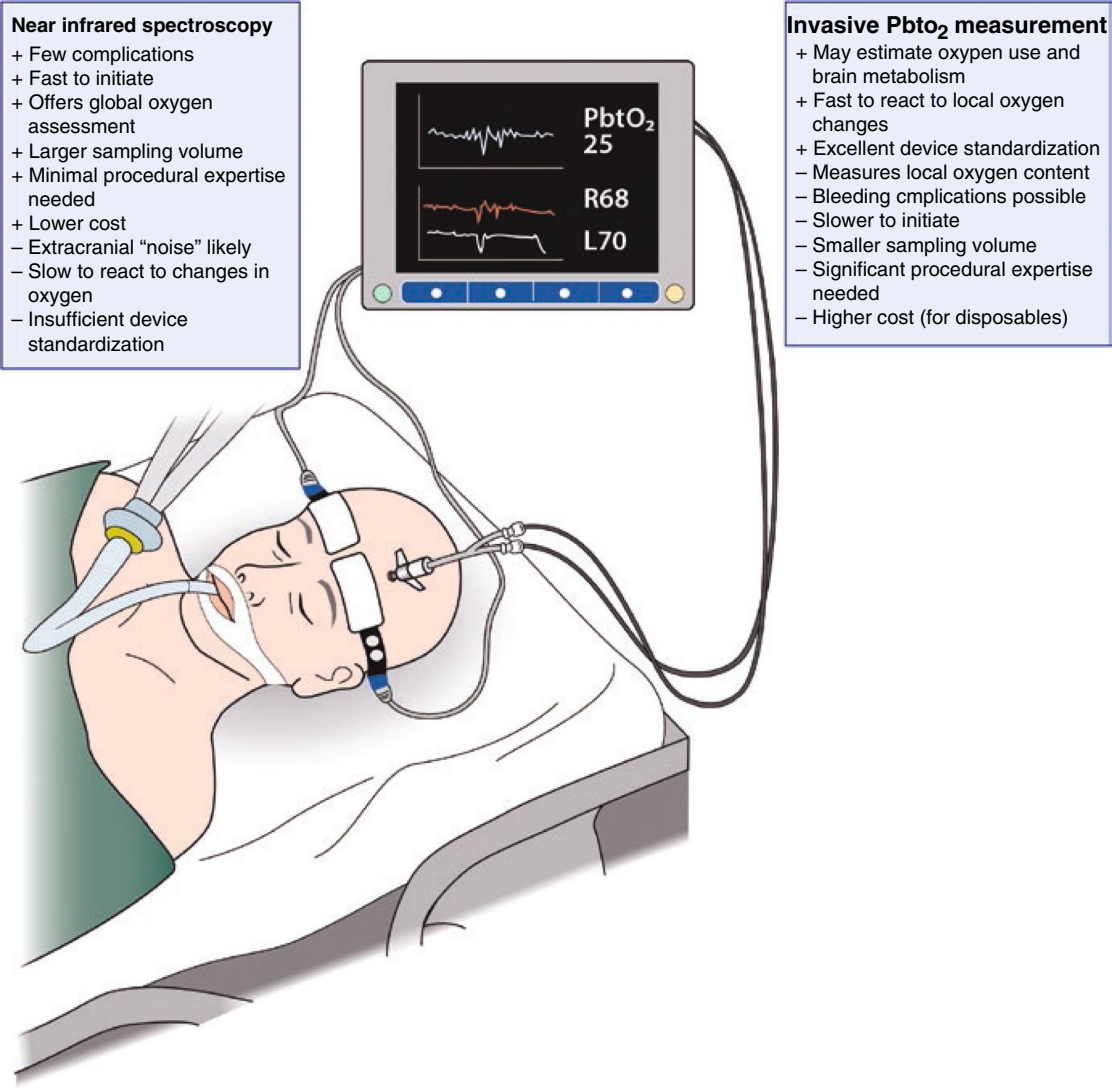
An overview of the advantages and disadvantages of one non-invasive and one invasive method used to monitor cerebral oxygenation in cardiac arrest patients

In this narrative review, we discuss the available means for monitoring the occurrence of brain ischemia in patients at risk of hypoxic ischemic brain injury. Specifically, we decided to review the evidence for non-invasive monitoring, using NIRS and invasive monitoring via the insertion of tissue oxygen monitors and jugular bulb catheters. These two approaches to monitoring brain oxygenation have different advantages and limitations (Fig. 1). We also discuss ways to modify cerebral oxygenation, with a special focus on MAP and blood carbon dioxide and oxygen levels.

## Cerebral oxygenation monitoring using NIRS

The level of oxygen in brain tissue is determined by the ratio between oxygen delivery and oxygen consumption, along with factors that influence the transfer of oxygen from the intravascular to the cellular compartment. The extent of hypoxic ischemic brain injury following cardiac arrest may be variably related to the degree and timing of aberrations in any or all of these variables. Importantly, the brain is able to maintain a relatively constant delivery of oxygen by maintaining CBF across a range of arterial blood pressure. This is referred to as cerebrovascular autoregulation [11]. In commercially available NIRS monitors, near infrared light is emitted from one diode—using at least two different wavelengths to assess oxyhemoglobin (HbO2) and total hemoglobin (Hb)—and received by another two diodes at separate distances. The latter feature enables the separation of light that has traversed the superficial (extracranial) versus deeper (intracranial, at a depth of 2–3 cm) tissues [12]. The NIRS sensors are commonly placed high on the temple in front of the hairline, thus illuminating the watershed area between the anterior and middle cerebral artery vascular territories. The rSO2 signal represents a ratio of HbO2/Hb. It is based on an assumption of fixed arterial and venous compartments, with the latter representing 70–75%, and is derived using proprietary algorithms that make it difficult to compare results obtained by different monitors. The inherent non-pulsatile nature of rSO2 means that it is, at best, a surrogate variable for CBF. The rSO2 is not just affected by anatomical confounders, such as the variable thickness of the extracranial tissues, the skull and the cerebrospinal fluid (CSF) area. It is also affected by dynamic factors, including anemia; by the patient’s acid-base status; by changes to the arteriovenous partitioning of blood; by tissue edema; and by progression of hypoxic ischemic brain injury to areas of non-metabolizing brain tissue. Generic to most clinical monitoring, rSO2 trends are more informative than absolute values. Yet it is unclear whether mean values, highest/lowest values or changes in values should be used as measures, and whether trends should be used to identify impending serious adverse events, to maintain a safety zone, or to trigger interventions. Concurrent changes in several variables—rather than in a single variable—may better reflect any underlying pathology; a growing number of reports of cerebrovascular autoregulation based on NIRS attests to an increasing interest in this approach. The physiological construct of using NIRS-derived rSO2 to monitor and potentially guide interventions against hypoxic ischemic brain injury entails the components of the rSO2 signal (e.g., blood transfusion, supplemental oxygen); the relation to oxygen delivery (e.g., supporting cardiac output and MAP, targeting optimal cerebrovascular autoregulation range); and the relation to oxygen consumption (e.g., targeted temperature management [TTM], analgosedation, seizure prophylaxis).

## Regional tissue oxygenation in hypoxic brain injury

While the use of rSO2, to inform on the quality of cardiopulmonary resuscitation (CPR) or to predict ROSC [13], supports the feasibility of NIRS in cardiac arrest and its potential to guide acute resuscitation, the main focus of this text is on post-resuscitation care. Multiple studies have been conducted, giving variable results regarding the possible differences in rSO2 values between patients with a good versus a poor functional outcome (Table 1). It appears biologically plausible that rSO2 values indicating that brain oxygen homeostasis has been maintained would be associated with survival and favorable neurological outcome. In an observational study of 28 cardiac arrest patients, rSO2 was lower following the initiation of hypothermia in non-survivors (n = 10) compared to survivors (n = 28) censored at hospital discharge [14]. Similar results were reported in 60 cardiac arrest patients, in which rSO2 during the first 40 h of intensive care unit (ICU) monitoring, including hypothermia and rewarming, was higher in patients with good outcomes (cerebral performance category [CPC] 1–2) compared to poor outcomes (CPC 3–5), both at ICU discharge and at 6 months, albeit with a large overlap in rSO2 values [15]. A larger prospective study (n = 107) of rSO2 during the first 48 h of ICU admission, including hypothermia and rewarming, and its association with outcome at 3 months reported statistically higher rSO2 in patients with good outcomes (CPC 1–2) compared to those with poor outcomes (CPC 3–5). Yet the study authors noted that the numerical differences were small and not conducive to a clinically useful discrimination of outcomes [16]. Based on data from the Japanese J-POP registry, an rSO2 > 40%—measured immediately upon arrival in the emergency department following cardiac arrest—was associated with favorable neurologic outcome at day 90 [17, 18]. A review of 22 observational studies, encompassing 2436 patients, corroborated the associations between increasing and higher rSO2 in the post-cardiac arrest period and favorable outcomes [19]. Meanwhile, several studies since the review—including 258 out-of-hospital cardiac arrest patients—have failed to demonstrate either a correlation or sufficient discriminative power for rSO2 and good versus poor outcomes [20–24], or have found it only in a specific range of initial rSO2 (between 41 and 60%) during TTM [25]. A recent review concluded that the clinical utility of monitoring rSO2 to prognosticate a favorable neurological outcome remains unclear [26]. Further clinical research is needed to establish the role of static versus dynamic rSO2 values; the cut-off values for correlations to patient-centered outcomes, including during different interventions for hypoxic ischemic brain injury, notably TTM; and the minimal duration of monitoring. It is also important to address the variability in reported rSO2 signals across different NIRS monitors [27] and overall cerebral tissue oxygenation [28].

**Table 1:** A selection of studies evaluating associations between near infrared spectroscopy (NIRS) measured and derived variables with outcome, in intensive care unit (ICU)-treated out-of-hospital cardiac arrest

First author [ref]	Year	Design	Number of patients	Type of cardiac arrest	Outcome	Principal finding
Meex [14]	2013	Observational study	28	CA patients treated with TTM	Functional outcome by CPC at hospital discharge	Decrease in rSO2 during induction of TTM. Lower rSO2 levels in patients with poor outcome
Storm [15]	2014	Observational study	60	OHCA and IHCA	Functional outcome at discharge by CPC	Higher NIRS values in patients with good outcome. An rSO2 below 50% appeared associated with poor outcome
Ameloot [33]	2015	Observational study	51	All types of CA	Functional outcome at 180 days by CPC	Disturbed autoregulation more common in patients with chronic hypertension. Time below an autoregulation-derived optimal MAP was negatively associated with outcome
Pham [24]	2015	Observational study	23	OHCA	Functional outcome at 90 days by CPC	No difference in rSO2 in patients, by outcome. Suggestion of disturbed autoregulation in poor outcome patients
Bougle [20]	2016	Observational study	43	OHCA treated with TTM	Functional outcome by CPC on hospital discharge	Mean rSO2 was not different, when indexed by outcome, but the lowest measured was lower in poor outcome patients
Genbrugge [16]	2016	Observational study	107	OHCA	Functional outcome at 180 days by CPC	Slightly higher rSO2 in patients with good outcome. No reliable threshold value was identified
Saritas [21]	2018	Observational study	25	OHCA patients	Functional outcome by CPC on hospital discharge	No difference in rSO2, in patients with good and poor outcome
Jakkula [23]	2019	*Post-hoc* analysis of interventional data	120	VF arrests with a cardiac cause	Six-month functional outcome by CPC and brain injury assessed with NSE	No association between the mean, median, lowest or highest NIRS value during the first 36 h of ICU care with outcome or the level of NSE at 48 h

*CA* cardiac arrest, *CPC* cerebral performance category, *IHCA* in-hospital cardiac arrest, *MAP* mean arterial pressure, *NSE* neuron specific enolase, *OHCA* out-of-hospital cardiac arrest, *rSO2* regional cerebral oxygen saturation, *TTM* targeted temperature management, *VF* ventricular fibrillation

## Cerebral oxygenation index in hypoxic brain injury

Monitoring of rSO2 has been extended into the assessment of cerebrovascular auto-regulation, by investigating the simultaneous correlation with MAP (time domain analysis) based on the premise that short-term fluctuations in rSO2 are predominantly determined by changes in CBF. The correlation index—the cerebral oximetry index (CO_x_)—may be used to assess cerebrovascular autoregulation, limits of autoregulation, and optimal MAP to support CBF [29–32]. In a prospective, observational study of 51 cardiac arrest patients monitored for the first 24 h of ICU admission during TTM at 33 °C, 35% demonstrated impaired and shifted autoregulation. A higher MAP (100 mmHg) was identified as supporting CBF, compared to patients with intact cerebrovascular autoregulation (85 mmHg). Mortality at 3 months was higher than for patients with preserved cerebrovascular autoregulation and the time spent below the optimal MAP was negatively correlated with survival [33]. In another prospective, observational study of 23 cardiac arrest patients undergoing TTM at 36 °C during the first 24 h and with active avoidance of pyrexia thereafter, intermittent monitoring during the first 3 days post-cardiac arrest demonstrated higher CO_x_ values. This was consistent with impaired cerebrovascular autoregulation in non-survivors, on each day and as an overall average, compared to survivors (all with CPC 1–2 at 3 months follow up) [24]. The optimal MAP was higher in non-survivors (107 mmHg), compared to survivors (66 mmHg). In a proof-of-concept study, continuous monitoring for a median of 30 h in 20 post-cardiac arrest patients managed with TTM 33–36 °C for the first 24 h was able to generate cerebrovascular autoregulation data in all patients. Of these, 15% demonstrated impaired autoregulation with the actual MAP ± 5 mmHg outside the identified optimal MAP in 50% of the monitored time. Increasing temperature was associated with an increased CO_x_, suggesting impaired cerebrovascular autoregulation—particularly above 38 °C [34]. A prospective study in three Canadian teaching hospital ICUs demonstrated the feasibility of capturing CO_x_ and deriving optimal MAP in a median of 97% and 71%, respectively, of data collected during a median monitoring time of 47.5 h [35].

A variable correlation between CO_x_ and the pressure reactivity index—a cerebrovascular autoregulation reference standard of intracranial pressure (ICP) versus MAP—has been reported [36]. This may not seem surprising, given the limitations of rSO2 that remain intrinsic to CO_x_. Furthermore, cerebrovascular autoregulation is far more complex than just a linear relation between CBF and MAP. It also includes other non-linear correlations, in particular with O2 and CO2, as well as significant heterogeneity across the cerebral vasculature and anatomical regions of the brain. Data from the COMACARE cohort are currently undergoing further investigation, focusing on CO_x_ with the important aspect of encompassing protocolized ranges of MAP, O2 and CO2 [37].

## Invasive monitoring of cerebral oxygenation

ICP monitoring and invasive oxygen, blood flow and microdialysis catheters have mainly been used for research purposes in the management of patients after cardiac arrest, and are not recommended for routine care [38]. The risk of complications, such as brain hemorrhage, is the main reason for this; these complications may be markedly increased in cardiac arrest patients, given the use of anticoagulants, anti-platelet agents and TTM. In addition, cardiac arrest patients may be less commonly cared for in units with neurosurgical expertise. The availability and incorporation of multimodal invasive neuromonitoring is thus limited in post-cardiac arrest management. Recently, the research group of Sekhon and colleagues completed a prospective observational study using invasive PbtO2 monitoring in hypoxic ischemic brain injury following cardiac arrest [9]. They established feasibility and, interestingly, demonstrated a significant burden (~ 40% of the monitoring duration) of brain hypoxia (PbtO2 < 20 mmHg) despite goal-oriented management to optimize PbtO2 [9]. They also established key relationships between the physiologic determinants of cerebral oxygen delivery and PbtO2. Specifically, significant linear relationships between PbtO2 with MAP and the cerebral perfusion pressure (CPP) were observed, across the cohort [9]. These data were subsequently followed up with a matched cohort study investigating clinical outcomes in patients with hypoxic ischemic brain injury managed using goal-directed care guided by invasive neuromonitoring, compared with the standard of care that did not include invasive neuromonitoring (Sekhon, personal communication, 2021). Although the clinical outcomes were significantly better in the invasive neuromonitoring group, significant limitations—including inherent biases, small sample size and study design—are important considerations when interpreting the findings. In the studies of invasive neuromonitoring to date, serious adverse events pertaining to the placement of invasive neuromonitoring have not been noted. However, the inherent risks associated with placement—namely, precipitating intracranial bleeding—are key considerations in invasive neuromonitoring. The reported rate of intracranial bleeding with invasive neuromonitoring is approximately 0.5–1% and the necessity for therapeutic hypothermia in post-cardiac arrest patients may increase this further. While noting the inherent limitations, these two studies provide feasibility and a path to studying the use of invasive neuromonitoring in select hypoxic ischemic brain injury cases, as a prospective method.

Jugular venous bulb oximetry is an alternative method of cerebral oxygen delivery and utilization monitoring. In this method, an intravascular catheter is placed retrograde into the dominant jugular vein and positioned at the level of the jugular bulb, to measure the oxygen saturation of Hb (SjvO2) as it exits the cerebral vasculature. Historically, hypoxic ischemic brain injury-related studies incorporating jugular venous bulb oximetry have focused on linking the absolute value of Hb saturation with clinical outcomes. Previous authors have shown that an increased SjvO2 or decreased oxygen extraction fraction, seen at the jugular bulb, is associated with worse outcomes and mortality [39]. Monitoring the metrics of brain oxygenation seems like an attractive therapeutic target to optimize. Yet the physiologic data garnered by both brain tissue oxygen and jugular venous bulb oximetry monitoring can provide insights into the underlying pathophysiologic phenotype that may be exhibited by individual patients with hypoxic ischemic brain injury. The approach of uniform cerebral oxygen delivery augmentation assumes that—once oxygen is delivered to the cerebral capillary bed—there is intact diffusion across the blood brain barrier and normal cellular oxygen utilization, culminating in neuronal aerobic metabolism. In other words, the necessary steps in the oxygen cascade encompass a coupling between cerebral oxygen delivery and diffusion, along with cellular utilization. It was recently shown that patients with hypoxic ischemic brain injury exhibit pathophysiologic phenotypes that are characterized by an uncoupling of these components of the oxygen cascade [40]. In a *post-hoc* analysis of invasive neuromonitoring in hypoxic ischemic brain injury, we characterized one subset of patients exhibiting diffusion limitation, wherein there was an uncoupling between cerebral oxygen delivery and diffusion into the brain parenchyma [41]. Conversely, the other phenotype was characterized by intact coupling between cerebral oxygen delivery and parenchymal diffusion [41].

To numerically quantify these phenotypes, the difference between the dissolved partial pressure of oxygen in the cerebral venous vasculature (PvO2) and the observed PbtO2 yields the PvO2-PbtO2 gradient. This represents the efficiency of oxygen diffusion into the parenchyma at the neurovascular unit [40]. When the patient is in a state of normal health, a reduction in cerebral oxygen delivery leads to increased oxygen extraction in the microvasculature and, hence, to a reduced PvO2-PbtO2 gradient. The inability to do so confirms a diffusion limitation; its detection is made possible by combining data points from simultaneous PbtO2 and jugular venous bulb oximetry (yielding the PvO2) monitoring [40]. Key future research must aim not just to incorporate monitoring of brain oxygenation in hypoxic ischemic brain injury, but also to use the characteristics of the cerebrovascular physiology in individual patients, to reconcile the underlying pathophysiologic processes at play and identify therapeutic targets.

## Interventions available for modifying cerebral oxygenation

Studies conducted in patients with TBI have shown that, by increasing the fraction of inspired oxygen used, the amount of oxygen measured in brain tissue is greatly increased [42]. In the COMACARE trial, the use of moderate hyperoxia significantly increased cerebral oxygenation—even in the setting of normal MAP—with-out any major increase in markers of brain injury [8, 43]. A recent meta-analysis of RCTs, on the other hand, suggested an association between worse patient outcomes in patients routinely treated with higher oxygen fractions after cardiac arrest [44]. Importantly, no study to date has included oxygen within a multimodal strategy for the alleviation of brain tissue hypoxia.

Mild hypercapnia appeared to increase cerebral oxygenation in two conducted pilot studies, but with a variable effect on the markers of brain injury [8, 45]. The TAME (Targeted Therapeutic Mild Hypercapnia after Resuscitated Cardiac Arrest) trial is currently underway, with more than 1300 patients randomized to date [46]. Conversely, hypocapnia decreased CBF and cerebral oxygenation, as measured with NIRS and jugular bulb monitoring in patients undergoing TTM at 33 °C [47]. Overall, the ultimate effect of cerebral oxygenation caused by the modification of CO2 concentrations is likely to depend, to a large degree, on whether or not the patient has increased ICP and cerebral edema. There are limited data, thus far, on whether this is a common clinical problem in cardiac arrest patients—especially those undergoing TTM.

It is currently unclear whether targeting higher MAP, as a routine measure, will also result in increased cerebral oxygenation in cardiac arrest. The COMACARE trial included patients resuscitated from out-of-hospital cardiac arrest with ventricular fibrillation (VF) as the initial rhythm and did not demonstrate any change in rSO2 with the higher MAP target. On the other hand, the Neuroprotect trial—which, in addition to increasing MAP, included the optimization of cardiac output with an inotrope and the use of packed red blood cell transfusions—showed increased rSO2 in the patients randomized to the higher MAP target. Interestingly, in a pooled analysis of a subset of patients with myocardial infarction and shock, the use of a higher MAP target alleviated myocardial injury [48].

Thus far, there is limited evidence on other means to improve brain oxygenation. Hb values of less than 10 g/dl have been associated with poor outcome in patients after cardiac arrest [49]. On the other hand, in the only RCT conducted in cardiac arrest patients that included maintaining Hb greater than 10 g/dl as an intervention, the need for a transfusion of packed red blood cells was uncommon [6]. The evidence on other means used to optimize cerebral oxygenation—using, for example, osmotherapy for increased ICP—lacks evidence in cardiac arrest patients [38].

## Conclusions and need for future studies

There is no doubt that measuring cerebral oxygenation, either non-invasively or invasively, is necessary to detect cases of occult and potentially modifiable ischemia. The utility of NIRS to monitor cerebral oxygenation following hypoxic ischemic brain injury is exceedingly attractive—given its non-invasive ease of operation, which provides a continuous, real-time signal. Ongoing and future research will ultimately need to show whether this technology is ‘making important what we can measure’ or, instead, measuring what is important. The use of invasive catheters provides more detailed data, including local brain blood flow and oxygen, as well as metabolism. It may well be that invasive catheters are superior at identifying the more occult, albeit local, instances of brain hypoxia.

With regard to available interventions, there is no doubt that—by modifying MAP, blood oxygen and carbon dioxide levels—brain oxygenation can be manipulated. Whether this results in improved oxygen utilization is less clear. The approach taken in TBI care with a multimodal approach to alleviate ischemia appears very interesting [50], but will no doubt be challenging to put into practice in the general cardiac arrest population. Until more evidence is available, we should aim to treat patients according to current guidelines that include targeting a MAP greater than 65 mmHg, normocapnia with a PaCO2 of 4.5–6.0 kPa, and a PaO2 of 10–13 kPa.

## Data Availability

Not applicable.
